# Physiological Adaptations of Tibetan Antelopes to High-Altitude Hypoxia: Insights from Pulmonary and Vasoactive Peptide Studies

**DOI:** 10.3390/ani16203260

**Published:** 2026-10-16

**Authors:** Ri-Li Ge, Lan Ma, Qing Ga, Hui Yang

**Affiliations:** 1 Research Center for High Altitude Medicine, Qinghai University, Xining 810016, China; geriligao@hotmail.com (R.-L.G.); judyml-325@163.com (L.M.); g-7@live.cn (Q.G.); 2 Medical College, Qinghai University, Xining 810016, China

**Keywords:** Tibetan antelope, high-altitude adaptation, hypoxic pulmonary vasoconstriction (HPV), ventilatory response, pulmonary morphology, mitochondria, lamellar bodies, veterinary physiology

## Abstract

The Qinghai–Tibetan Plateau is characterized by persistent hypobaric hypoxia, and Tibetan antelopes (*Pantholops hodgsonii*) exhibit exceptional athletic performance under extreme oxygen-deficient conditions. This study conducted a comparative analysis of pulmonary hemodynamics, ventilatory regulation, vasoactive peptides, and cardiopulmonary ultrastructure among 10 Tibetan antelopes, 10 high-altitude sheep, and 8 low-altitude sheep under normoxia, mild hypoxia, and severe hypoxia. The findings revealed that Tibetan antelopes possess a lower baseline mean pulmonary arterial pressure (mPAP) compared to high-altitude sheep, with values similar to those of low-altitude sheep. Under hypoxic conditions, antelopes experienced a mild increase in mPAP, whereas sheep exhibited significant vasoconstriction. Additionally, antelopes demonstrated a reduced hypoxic ventilatory response, in contrast to sheep, which increased total ventilation. Antelopes also had lower levels of plasma endothelin-1, nitric oxide synthase, arterial oxygen pressure, and blood oxygen saturation, with no significant differences observed in blood pH, carbon dioxide pressure, and lactate levels among the groups. Ultrastructural analyses indicated that Tibetan antelopes possess a higher density of alveolar lamellar bodies and myocardial mitochondria. These traits minimize energy use during breathing, prevent pulmonary hypertension, and maintain excellent movement ability in high-altitude, low-oxygen environments.

## Simple Summary

The Qinghai–Tibet Plateau’s high altitude and low oxygen challenge most animals, but Tibetan antelopes excel in endurance and speed. To explore their adaptations, we compared ten Tibetan antelopes with sheep adapted to high and low altitudes. Subjects were exposed to normal air and two hypoxic levels using face masks to simulate higher altitudes. We assessed pulmonary arterial pressure, respiratory rate, blood oxygen saturation, and examined lung and heart tissues. Results showed Tibetan antelopes maintained stable pulmonary arterial pressure under hypoxia, unlike sheep, whose blood vessels constricted. Antelopes also had a reduced ventilatory response, conserving energy, and lower levels of vasoconstrictive agents. Microscopic analysis showed enhanced tissue adaptations. These adaptations allow Tibetan antelopes to manage oxygen scarcity, prevent pulmonary hypertension, and sustain physical activity. Understanding these adaptations may aid in developing health protection strategies.

## 1. Introduction

The Tibetan Plateau, characterized by an average elevation exceeding 4000 m above sea level, constitutes one of the most extreme terrestrial environments on Earth [1]. This region is marked by hypobaric hypoxia, intense solar radiation, significant diurnal temperature variations, and extreme cold. While the present study focuses on hypoxic adaptation, we acknowledge that cold stress represents an additional critical environmental factor, and Tibetan antelopes possess thick wool and specialized metabolic traits to cope with thermal challenges [1,2,3]. The interaction between cold and hypoxic adaptation warrants further investigation. Among the diverse fauna inhabiting this challenging environment, the Tibetan antelope (*Pantholops hodgsonii*) emerges as a particularly noteworthy endemic species. Despite the severe oxygen limitations at high altitudes—where the ambient oxygen partial pressure is approximately 60% of that at sea level—Tibetan antelopes exhibit extraordinary athletic capabilities. They are capable of sprinting at speeds exceeding 80 km per hour and undertake extensive seasonal migrations across the plateau [1,2,3]. This exceptional locomotor capacity in a highly hypoxic environment has long intrigued comparative physiologists and evolutionary biologists, prompting fundamental investigations into the mechanisms that facilitate such high-altitude tolerance.

Research efforts aimed at elucidating the mechanisms underlying hypoxia adaptation in Tibetan antelopes have predominantly concentrated on molecular and genomic dimensions. Transcriptomic analyses of pulmonary and cardiac muscle tissues have identified distinct expression profiles, with numerous genes associated with energy metabolism undergoing rapid adaptive evolution in this species [4,5]. Recent biochemical investigations have further demonstrated an adaptive enhancement in blood oxygen affinity, attributed to the truncation of ancestral globin gene ontogeny. This truncation results in the complete replacement of the lower-affinity adult hemoglobin isoform, typical of other bovids, with a high-affinity juvenile hemoglobin isoform [6]. Additionally, cardiac morphometric analyses have indicated that Tibetan antelope hearts exhibit higher heart-weight-to-body-weight ratios, increased contractility under hypoxic conditions, and elevated expression of key genes involved in the regulation of contractility and hypertrophy, compared to Tibetan sheep [7]. These molecular and structural insights have significantly advanced our comprehension of the mechanisms by which Tibetan antelopes adapt to chronic hypoxia.

Despite advancements in our understanding, a significant gap persists in elucidating the in vivo integrative physiological mechanisms governing oxygen transport in this species, particularly concerning pulmonary circulation and ventilatory regulation. Hypoxia profoundly impacts two critical components of the oxygen cascade: pulmonary vascular tone, which determines right ventricular afterload and ventilation-perfusion matching, and ventilatory drive, which directly influences arterial oxygenation. In most lowland mammals, acute hypoxia induces hypoxic pulmonary vasoconstriction (HPV)—a homeostatic mechanism that redirects blood flow from poorly ventilated to well-ventilated lung regions—and triggers a robust hypoxic ventilatory response (HVR) that enhances alveolar ventilation to improve oxygen uptake [8,9]. However, these physiological responses may become maladaptive during prolonged exposure to high-altitude environments. While acute HPV is a beneficial homeostatic response lasting minutes to hours, sustained hypoxic pulmonary vasoconstriction and subsequent pulmonary vascular remodeling under chronic hypoxia can lead to pulmonary hypertension, right ventricular hypertrophy, and ultimately high-altitude heart disease [10,11]. An exaggerated HVR can impose a significant metabolic burden and disrupt acid-base homeostasis [10,11].

Certain native species residing in high-altitude environments have distinctly evolved an attenuated phenotype concerning specific physiological responses, which appears to confer an adaptive advantage. For example, the plateau pika (*Ochotona curzoniae*) has nearly lost the mechanism of HPV, exhibiting a minimal pulmonary pressor response to hypoxia and possessing thin-walled pulmonary arterioles [12,13]. Similarly, Tibetan humans demonstrate reduced HPV and a blunted HVR compared to lowland populations [14,15]. Among domesticated ruminants, Tibetan sheep—an indigenous breed of the plateau—exhibit elevated hemoglobin concentrations and altered blood-gas variables relative to lowland sheep. However, their pulmonary hemodynamic responses to hypoxia have not been systematically characterized in direct comparison with truly wild high-altitude specialists [16,17]. It remains uncertain whether Tibetan antelopes, as non-speciated wild bovids that have inhabited the plateau for millions of years, exhibit a similar attenuation of HPV and/or HVR, and, if so, what morphological substrates in the lung and heart underlie such functional adaptations. At the molecular level, high-altitude adaptation involves multiple interconnected pathways. Hypoxia-inducible factor (HIF) pathway genes, including *EPAS1* (encoding HIF-2α) and *EGLN1* (encoding prolyl hydroxylase 2), regulate the primary transcriptional response to oxygen deprivation [4,5,9]. Energy metabolism genes such as *PPARA* and *PPARGC1A* control fatty acid oxidation and mitochondrial biogenesis, supporting sustained aerobic ATP production under hypoxia [4,10]. The globin gene cluster exhibits adaptive truncation in Tibetan antelopes, resulting in the persistence of high-affinity fetal hemoglobin (HBG) into adulthood, thereby enhancing oxygen transport efficiency [6]. Additionally, vascular tone and remodeling regulators—including *EDN1*, *NOS2*, and *VEGFA*—modulate endothelial function and pulmonary vascular structure [5,18]. These molecular adaptations provide the mechanistic foundation for the integrative physiological phenotypes examined in the present study.

The interaction between pulmonary circulation and cardiac ultrastructure is crucial for resilience to hypoxic stress. In lowland mammals, prolonged exposure to hypoxia generally results in the thickening of the pulmonary arterial media, an increase in smooth muscle content, and the development of pulmonary hypertension, which is associated with remodeling of the right ventricle [8,13]. In contrast, animals native to high-altitude environments often display structural adaptations that counteract these pathological changes. These adaptations include thinner alveolar septa to reduce diffusion distance, a higher density of tissue microvasculature, and ultrastructural modifications in alveolar type II cells—such as lamellar bodies that regulate surfactant production and enhance gas exchange efficiency—as well as in cardiomyocytes, which are characterized by an abundance of mitochondria that support aerobic metabolism [9,12,18,19,20]. Nonetheless, there is a significant deficiency in comprehensive comparative data that integrate pulmonary hemodynamics, ventilatory control, circulating vasoactive factors, and the microstructure of pulmonary and cardiac tissues in Tibetan antelopes, particularly in comparison to suitable altitude-matched and lowland control animals. Such integrated comparative physiological data are essential for elucidating the evolutionary mechanisms underlying high-altitude adaptation in wild bovids and for informing conservation strategies for this endangered species in the face of ongoing environmental changes on the Tibetan Plateau.

To fill knowledge gaps, we compared Tibetan antelopes, high-altitude plateau sheep, and low-altitude sheep in a study focusing on: (1) baseline mean pulmonary arterial pressure (mPAP) and hypoxic pulmonary vasoconstriction (HPV) during hypoxia; (2) ventilatory adaptations in normoxic and hypoxic conditions; (3) levels of endothelin-1 (ET-1) and nitric oxide synthase (NOS), key regulators of pulmonary vascular tone; and (4) pulmonary and cardiac ultrastructure, especially alveolar lamellar bodies and myocardial mitochondria. These findings helped us propose a physiological model of high-altitude adaptation in Tibetan antelopes. We hypothesized that Tibetan antelopes, unlike both high-altitude and low-altitude sheep, would exhibit a reduced hypoxic pulmonary vasoconstriction (HPV) response and a diminished hypoxic ventilatory response (HVR). Furthermore, we expected to observe unique pulmonary and cardiac morphological features, such as a high density of alveolar lamellar bodies and myocardial mitochondria. These adaptations are proposed to enhance oxygen delivery and utilization under extreme hypobaric hypoxia conditions, suggesting evolutionary optimization over millennia through natural selection on the Qinghai–Tibetan Plateau.

## 2. Materials and Methods

### 2.1. Study Animals and Ethical Approval

All experimental protocols were approved by the Animal Ethics Committee of Qinghai University Medical College (Approval No.: QUMC-AEC-2018-027) and followed the NIH Guide for the Care and Use of Laboratory Animals (8th ed., 2011). The study also adhered to the ARRIVE guidelines 2.0.

Only adult males were included to eliminate potential confounding effects of sex hormones (e.g., estrogen and progesterone) on cardiopulmonary hemodynamics and ventilatory control, and to avoid variability associated with estrous cycles, which are known to influence vascular tone and chemoreceptor sensitivity in ruminants. A total of 28 healthy adult male specimens were utilized in this study, comprising 10 Tibetan antelopes (*Pantholops hodgsonii*) sourced from the Kekexili region, 10 high-altitude sheep (*Ovis aries*) from Maduo County, and 8 low-altitude sheep (*Ovis aries*) from a farm located in Pingliang, Gansu Province, at an altitude of 1300–1500 m. All subjects were aged between 3 and 5 years and exhibited no clinical signs of disease. To minimize genetic variability, the sheep were obtained from single commercial flocks. The Tibetan antelopes were derived from a single wild population in Kekexili, with no siblings included in the sample.

Low-altitude sheep underwent a 14-day acclimatization period at the experimental facility in Golmud City (2800 m). In contrast, Tibetan antelopes and high-altitude sheep, both indigenous to altitudes exceeding 4000 m, were subject to a 5-day acclimatization period at the same location, constrained by permit limitations. This discrepancy in acclimatization durations constitutes a significant limitation: the abbreviated 5-day period for Tibetan antelopes and high-altitude sheep may be inadequate for the complete stabilization of hematological parameters, particularly hemoglobin (Hb) and hematocrit (Hct). Additionally, high-altitude sheep may have undergone partial de-acclimatization from their native altitudes, potentially leading to an underestimation of the true physiological differences between high-altitude sheep and Tibetan antelopes under fully native conditions. Throughout the acclimatization phase, all animals were observed bi-daily for appetite, behavior, and fecal consistency, with no signs of illness or distress detected. Notably, all Tibetan antelopes maintained normal food intake, stable body weight, and typical fecal pellet morphology during the 5-day period, with no indications of capture myopathy, such as myoglobinuria, recumbency, or muscle rigidity. The animals were housed individually, provided with ad libitum access to water, and fed a diet of alfalfa hay (1.5 kg/day) supplemented with 200 g/day of mixed concentrate. No mortality or severe distress was recorded.

### 2.2. Experimental Design and Hypoxic Challenges

**Figure 1:**
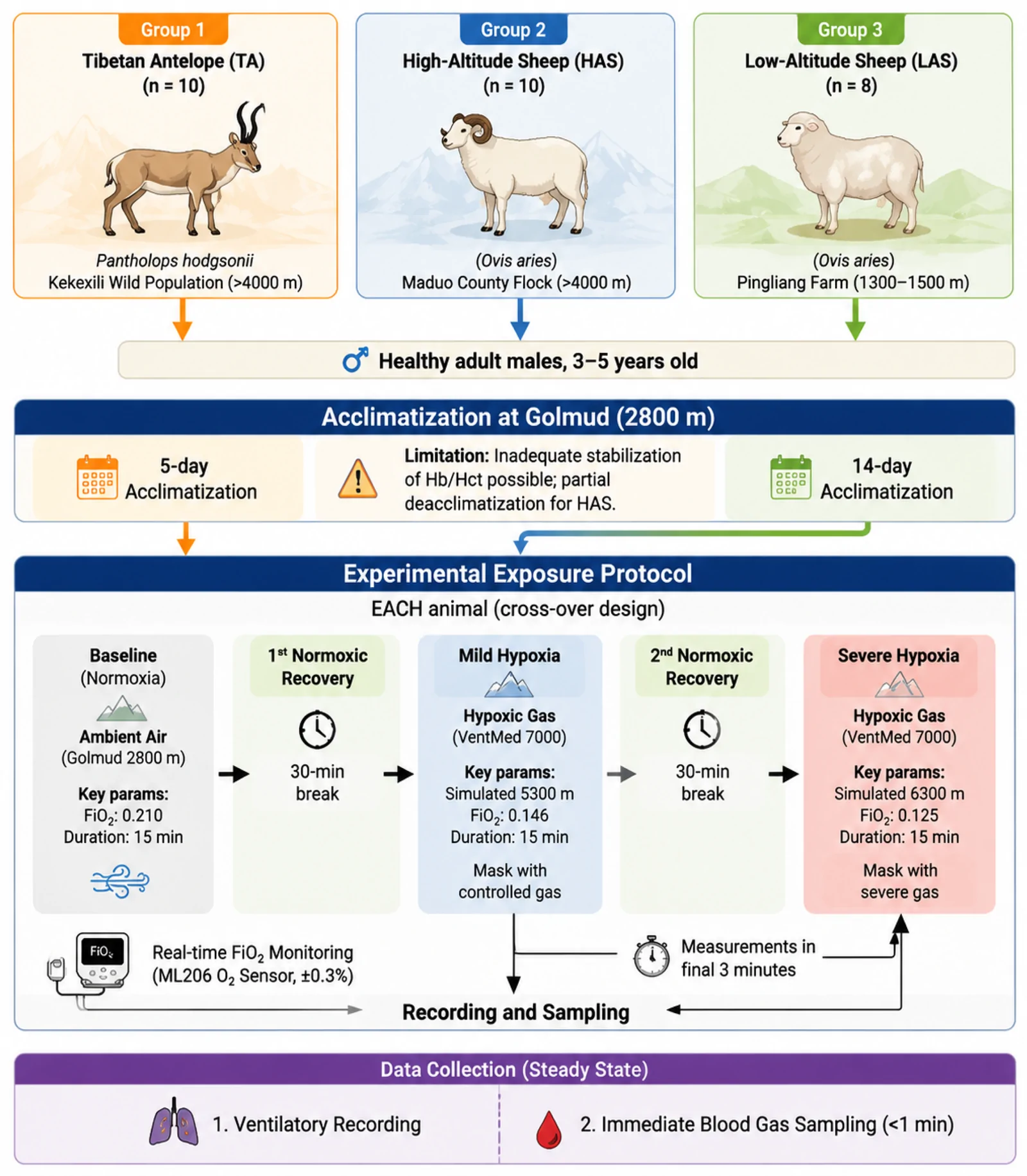
Schematic diagram of the whole experimental workflow, including animal acclimatization, anesthesia, graded hypoxic stimulation, multi-index recording, blood sampling, tissue collection and ultrastructural detection.

The animals were categorized into three distinct groups: Tibetan antelope (TA, *n* = 10), high-altitude sheep (HAS, *n* = 10), and low-altitude sheep (LAS, *n* = 8). Each subject underwent exposure to three consecutive conditions—baseline, mild hypoxia, and severe hypoxia—with a 30 min normoxic recovery period interspersed between each exposure. The baseline condition (normoxia) was characterized by ambient air at Golmud (2800 m, FiO_2_ 0.210, barometric pressure approximately 530 mmHg, inspired PO_2_ approximately 111 mmHg). The mild hypoxia condition involved an FiO_2_ of 0.146 for a duration of 15 min, simulating an altitude of approximately 5300 m. The severe hypoxia condition entailed an FiO_2_ of 0.125 for 15 min, simulating an altitude of approximately 6300 m. Hypoxic gases were administered through a tightly fitting face mask connected to a gas mixing system (VentMed 7000, Beijing Aeonmed Co., Ltd., Beijing, China). The actual FiO_2_ within the mask was continuously monitored using an O_2_ sensor (ML206, ADInstruments, Dunedin, New Zealand) and was maintained within ±0.3% of the intended value. Measurements were recorded during the final 3 min of each condition to ensure a steady state, and blood gas sampling was conducted immediately following the ventilatory recording period, with an interval of less than 1 min. A summary of the experimental workflow is presented in Figure 1.

### 2.3. Pulmonary Hemodynamics and Anesthesia Protocol

In order to mitigate anesthesia-induced cardiopulmonary depression, animals were administered ketamine (3.5 mg/kg, intramuscularly) and diazepam (0.1 mg/kg, intramuscularly) as pre-anesthetic medication. Atropine sulfate (0.02 mg/kg, intramuscularly) was administered five minutes prior to induction to avert bradycardia. Anesthesia was maintained using 1.2–1.5% isoflurane in oxygen delivered via a face mask. The animals were positioned on a heating pad set to 38.0 ± 0.5 °C in left lateral recumbency to preserve normothermia. Physiological parameters including heart rate, respiratory rate, end-tidal CO_2_ (ETCO_2_), peripheral oxygen saturation (SpO_2_), and rectal temperature were continuously monitored using a Datex-Ohmeda S/5 monitor (Helsinki, Finland). No arrhythmias or significant hypoxemia (SpO_2_ < 85%) were observed during catheter placement. Hemodynamic measurements commenced following a 30-min stabilization period, once heart rate and ETCO_2_ had stabilized. Subsequent to local infiltration with 2% lidocaine, a 7-Fr Swan–Ganz catheter (Edwards Lifesciences, Irvine, CA, USA) was inserted via the right external jugular vein and advanced into the pulmonary artery under pressure waveform guidance. Continuous pressure waveform recordings were obtained using a 16-channel PowerLab data-acquisition system (ADInstruments, Sydney, Australia) at a sampling frequency of 200 Hz. The catheter’s final position was verified through fluoroscopic imaging.

The hemodynamic parameters evaluated in this study comprised mean pulmonary arterial pressure (mPAP), pulmonary capillary wedge pressure (PCWP), cardiac output (CO), and heart rate (HR). Cardiac output was quantified using the thermodilution technique, which entailed the administration of three successive 5-mL bolus injections of ice-cold 5% dextrose solution. Measurements were considered valid when the variation among the three readings was less than 10%, and the mean of these readings was utilized for subsequent analyses. Pulmonary vascular resistance (PVR) was calculated using the formula: PVR = (mPAP − PCWP)/CO × 80 (dyn·s·cm^−5^), where PVR is expressed in dyn·s·cm^−5^, mPAP and PCWP are measured in mmHg, and CO is measured in L/min.

### 2.4. Ventilatory Measurements

Tidal volume (VT) and respiratory frequency (fR) were assessed utilizing a pneumotachograph (Hans Rudolph, Inc., Kansas City, MO, USA) affixed to a face mask. The mask was secured with a silicone rim, and potential leaks were evaluated by sampling CO_2_ at the mask’s periphery. Flow signals were integrated in real-time using LabChart 8.0 (ADInstruments) to determine VT, adjusted to body temperature, pressure, and saturation (BTPS) conditions. Minute ventilation (VE) was computed as the product of VT and fR (L/min, BTPS). For analysis, mean values from the final 3 min of a 5-min steady-state recording were utilized.

### 2.5. Arterial Blood Gas and Lactate Analysis

Under local anesthesia, a 22-gauge catheter was inserted into the auricular artery. Following the completion of each experimental condition, immediately after the ventilatory recording, 1.0 mL of blood was drawn anaerobically into a heparinized syringe and analyzed within five minutes using a GEM Premier 3500 analyzer (Instrumentation Laboratory, Bedford, MA, USA). The parameters measured included partial pressures of oxygen (PaO_2_) and carbon dioxide (PaCO_2_), oxygen saturation (SaO_2_), pH, hemoglobin concentration (Hb), hematocrit (Hct), and lactate levels (Lac). The analyzer underwent daily calibration. All measurements were adjusted for core body temperature, as determined by a rectal thermometer with an accuracy of ±0.1 °C. The mean rectal temperatures recorded during the measurements were 38.4 ± 0.3 °C for the TA group, 38.6 ± 0.4 °C for the HAS group, and 38.5 ± 0.3 °C for the LAS group, with no statistically significant differences observed among the groups (*p* = 0.42).

### 2.6. Vasoactive Peptide Assays

Venous blood (5 mL) was collected into EDTA tubes and subsequently centrifuged at 3000× *g* for 10 min at 4 °C. The plasma obtained was stored at −80 °C. Endothelin-1 (*ET-1*) levels were quantified using an enzyme-linked immunosorbent assay (ELISA) provided by Cloud-Clone Corporation, Wuhan, China (Catalog No. SEA071Bo), with a detection range of 15.6–1000 pg/mL and intra- and inter-assay coefficients of variation (CV) of less than 8% and 10%, respectively. Nitric oxide synthase (*NOS*) activity was assessed via a colorimetric assay from the Nanjing Jiancheng Bioengineering Institute, Nanjing, China (Catalog No. A014-2), and the results were expressed as units per milligram of protein, with protein concentration determined by the bicinchoninic acid (BCA) method. All assays were conducted in duplicate.

### 2.7. Pulmonary and Myocardial Ultrastructure

After physiological assessments, the animals were euthanized with sodium pentobarbital (120 mg/kg) under deep anesthesia, administered intravenously at 1 mL/s until reflexes ceased. An additional 60 mg/kg dose ensured cardiac arrest, confirmed by the absence of heart activity and breathing, following AVMA guidelines. During necropsy, the lungs and heart showed no signs of pulmonary edema, consolidation, right or left ventricular hypertrophy, or other abnormalities. No parasitic nodules, abscesses, or evidence of pulmonary echinococcosis or dictyocaulosis were found. Tissue samples were procured from the right middle lung lobe and the free wall of the left ventricle. The left ventricle was selected to assess myocardial oxidative capacity supporting systemic oxygen delivery during high-speed locomotion, which is a key aspect of Tibetan antelope adaptation. The left ventricle was selected to assess myocardial oxidative capacity supporting systemic oxygen delivery during high-speed locomotion, which is a key aspect of Tibetan antelope adaptation. These samples were immediately fixed in 2.5% glutaraldehyde, subsequently post-fixed in 1% osmium tetroxide, dehydrated through a graded series of solvents, and embedded in Epon 812 resin. Ultrathin sections, measuring 70–80 nm, were prepared and stained with uranyl acetate and lead citrate prior to examination via transmission electron microscopy. The quantification of lamellar bodies within alveolar type II epithelial cells was conducted in 10 randomly selected high-magnification fields per animal at 15,000× magnification. The results were reported as the number of lamellar bodies per 100 μm^2^ of alveolar septal area to account for variations in alveolar expansion. Myocardial mitochondrial density was assessed in five randomly selected fields per animal at 20,000× magnification using ImageJ software (version 1.53c; National Institutes of Health, Bethesda, MD, USA) and was expressed as the percentage of the cytoplasmic area occupied by mitochondria. All morphometric analyses were conducted by a veterinary pathologist who was blinded to the experimental group assignments.

### 2.8. Statistical Analysis

The data are expressed as mean ± standard deviation (SD). The assumptions of normality and homogeneity of variance were evaluated using the Shapiro–Wilk test and Levene’s test, respectively. Endothelin-1 (*ET-1*) and nitric oxide synthase (NOS) data were log-transformed to achieve normality prior to conducting a one-way analysis of variance (ANOVA) for baseline comparisons. The magnitudes of response (ΔmPAP, ΔVE) were analyzed across groups using linear mixed models (LMM), incorporating group, condition, and group-by-condition interaction as fixed effects, with individual animals treated as random intercepts to account for repeated measures within subjects. Post hoc pairwise comparisons were conducted with Tukey’s adjustment for multiple testing. For baseline comparisons among the three groups, one-way ANOVA with Tukey’s Honestly Significant Difference (HSD) test was applied when variances were homogeneous, while Welch’s ANOVA with Games–Howell post hoc test was used when variances were unequal. Exploratory Pearson correlation analyses were conducted to examine the relationship between plasma levels of *ET-1/NOS* and ΔmPAP across all animals, thereby assessing the association between vasoactive peptides and pulmonary pressor responses.

All *p*-values are two-tailed, and *p* < 0.05 was considered significant. A priori power analysis for ΔmPAP (Cohen’s d = 0.9, α = 0.05, power = 0.80) required 24 animals; we included 28 to account for potential technical issues. However, the LAS subgroup (*n* = 8) is at the lower bound of the calculated per-group requirement, which may reduce statistical power for secondary morphometric analyses and interaction effects. Given the wild origin of TA with inherently higher inter-individual variability (as reflected in larger SDs for several parameters), we acknowledge that the study may be underpowered to detect subtle differences within the TA group. Statistical analyses were performed using SPSS 25.0 and R 4.2 (lme4 package), and graphs were generated with GraphPad Prism 9.0.

## 3. Results

### 3.1. General Characteristics and Hematological Parameters

Table 1 delineates the general characteristics and baseline hematological parameters of the study groups. The three groups exhibited comparable body weight and age. Notably, the TA group demonstrated a significantly elevated lung weight-to-body weight ratio in comparison to both sheep groups (*p* < 0.01). The red blood cell (RBC) count and hemoglobin levels in the TA group were analogous to those in the LAS group (*p* > 0.05), yet were lower than those in the HAS group (*p* < 0.05), with hematocrit values exhibiting a similar trend. Plasma levels of endothelin-1 (ET-1) and nitric oxide synthase (NOS) were markedly reduced in the TA group relative to both HAS and LAS groups (*p* < 0.001), with ET-1 levels also being lower in the LAS group compared to the HAS group (*p* < 0.05). These findings imply that TA substantially mitigates systemic endothelial activation and inflammatory stress, as evidenced by decreased ET-1 and NOS levels. Conversely, HAS appears to afford superior pulmonary protection but is associated with significant hemoconcentration and endothelial complications. LAS confers pulmonary benefits comparable to HAS but with a reduced incidence of vascular side effects.

Table 2 presents the baseline arterial blood gas values. No significant differences were observed in pH, PaCO_2_, or lactate levels among the groups. Nevertheless, the TA group exhibited significantly lower PaO_2_ and SaO_2_ levels compared to the sheep groups (*p* < 0.001), suggesting diminished baseline arterial oxygenation, likely attributable to their higher altitude habitat. This finding underscores the necessity to investigate the functional implications of their reduced ventilatory and vascular responses. Notably, the normal lactate levels in the TA group imply that any tissue hypoxia was insufficient to induce anaerobic metabolism under resting, anesthetized conditions.

### 3.2. Ventilatory Response to Hypoxia

**Figure 2:**
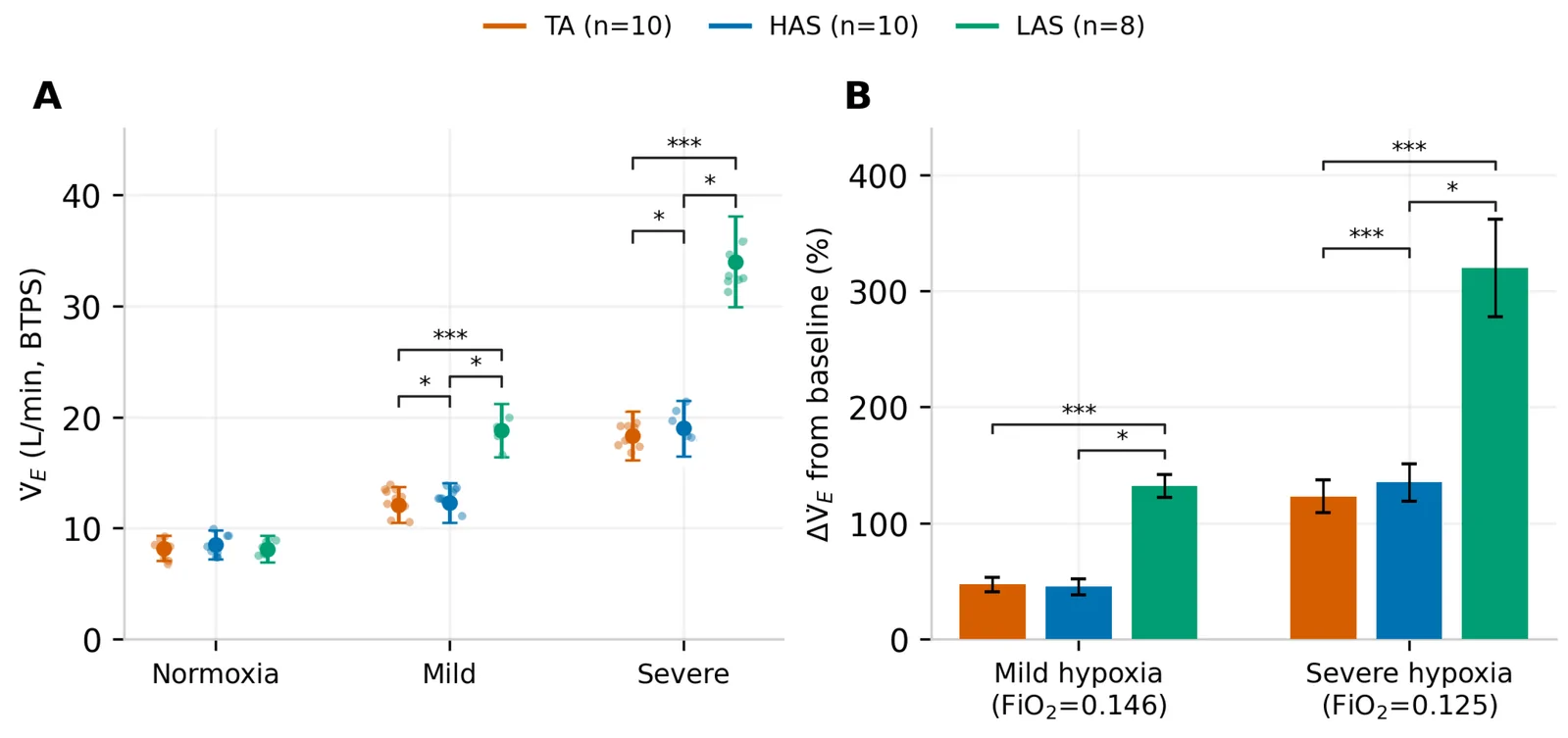
Illustrates the ventilatory responses to graded hypoxia in Tibetan antelope (TA), high-altitude sheep (HAS), and low-altitude sheep (LAS). Panel (**A**) depicts the absolute minute ventilation (VE, L/min, BTPS) recorded under normoxic conditions (FiO_2_ = 0.210), mild hypoxia (FiO_2_ = 0.146), and severe hypoxia (FiO_2_ = 0.125) for TA (*n* = 10), HAS (*n* = 10), and LAS (*n* = 8). The data are expressed as mean ± standard deviation (SD), with individual data points superimposed. Panel (**B**) shows the relative increase in VE from the normoxic baseline (%) under conditions of mild and severe hypoxia, with data presented as mean ± standard error of the mean (SEM). Differences between groups at each oxygen level were evaluated using linear mixed models, with Tukey’s post-hoc adjustment applied for multiple comparisons. Statistical significance is denoted as follows: * *p* < 0.05, *** *p* < 0.001 between the specified groups. BTPS refers to body temperature, pressure, and saturation conditions; FiO_2_ denotes the fraction of inspired oxygen.

Figure 2A,B provides a summary of minute ventilation (VE) across the three oxygen conditions, while Figure S1 displays representative respiratory waveforms. Under normoxic conditions (FiO_2_ = 0.210), baseline VE was similar across the three groups: TA (8.2 ± 1.1 L/min), HAS (8.5 ± 1.3 L/min), and LAS (8.1 ± 1.2 L/min), with no statistically significant differences (all *p* > 0.05). During graded hypoxia, a significant increase in VE from the normoxic baseline was observed in both sheep groups, HAS and LAS (*p* < 0.01 and *p* < 0.001, respectively), whereas the TA group did not exhibit a statistically significant increase (*p* > 0.05). In terms of relative changes, VE increased from baseline by 47% and 123% in the TA group, 45% and 135% in the HAS group, and 132% and 320% in the LAS group under mild (FiO_2_ = 0.146) and severe (FiO_2_ = 0.125) hypoxic conditions, respectively (Figure 2B). Comparative analyses between groups indicated that the hypoxic ventilatory response was significantly attenuated in the TA group compared to both sheep groups. At both levels of hypoxia, VE was significantly lower in the TA group than in the HAS and LAS groups (*p* < 0.001 for both comparisons; Figure 2A), and the relative increase from baseline was also significantly smaller in the TA group compared to the LAS (*p* < 0.001) and HAS groups (*p* < 0.001; Figure 2B). Moreover, the low-altitude sheep (LAS) demonstrated a markedly greater ventilatory response compared to the high-altitude sheep (HAS) under conditions of severe hypoxia (*p* < 0.05; Figure 2A), with a comparable pattern noted for the relative increase (*p* < 0.05; Figure 2B). These findings suggest that Tibetan antelope (TA) exhibit a diminished hypoxic ventilatory response (HVR) in comparison to both high- and low-altitude sheep, while the LAS exhibit the most robust ventilatory compensation among the groups studied.

### 3.3. Pulmonary Hemodynamics

**Figure 3:**
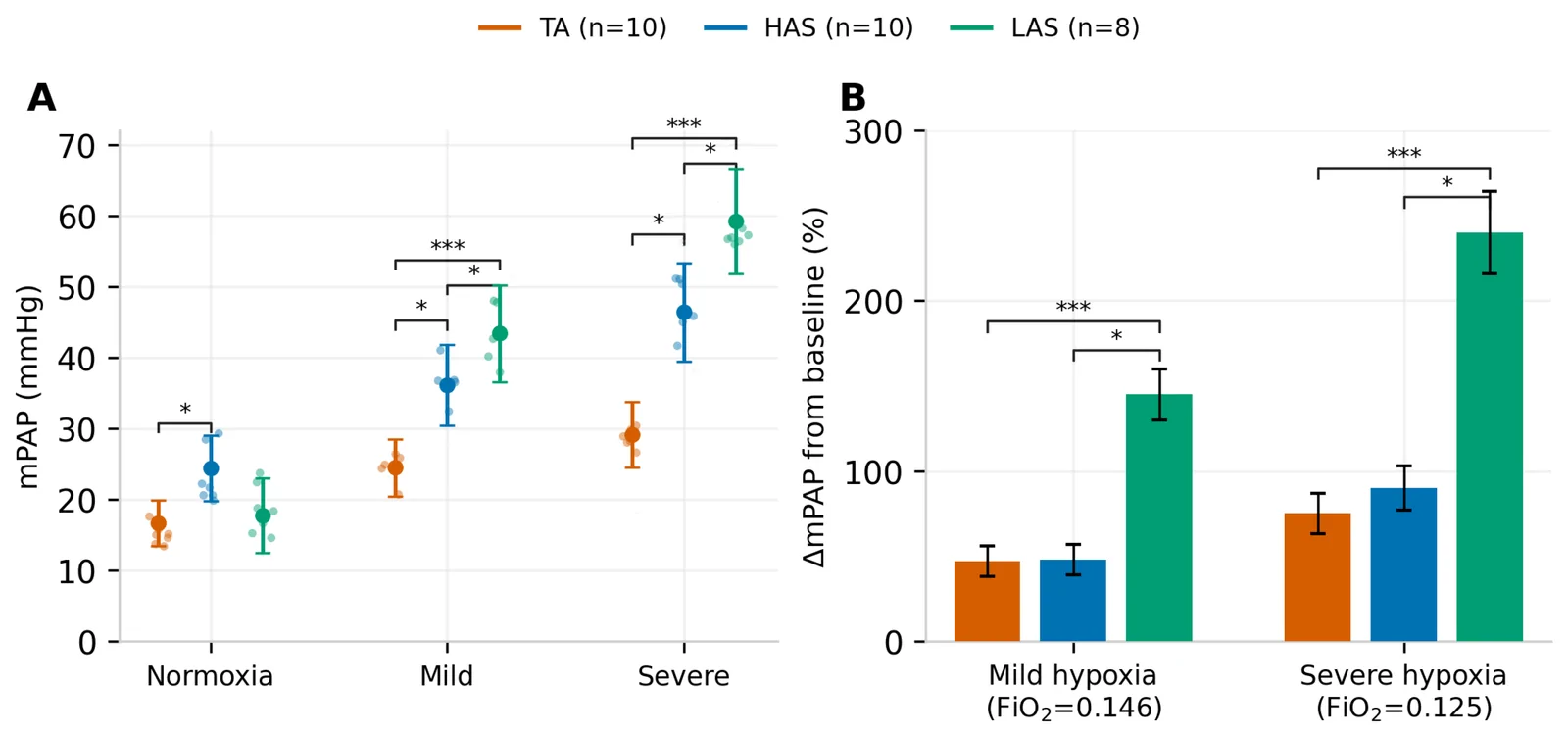
The pulmonary hemodynamic responses to graded hypoxia in Tibetan antelope (TA), high-altitude sheep (HAS), and low-altitude sheep (LAS). Panel (**A**) depicts the absolute mean pulmonary arterial pressure (mPAP, measured in mmHg) under normoxic conditions (FiO_2_ = 0.210), mild hypoxia (FiO_2_ = 0.146), and severe hypoxia (FiO_2_ = 0.125) in TA (*n* = 10), HAS (*n* = 10), and LAS (*n* = 8). The data are expressed as mean ± standard deviation (SD), with individual data points overlaid. Panel (**B**) shows the relative increase in mPAP from the normoxic baseline (%) under conditions of mild and severe hypoxia, presented as mean ± standard error of the mean (SEM; *n* = 6 per group). Differences between groups at each oxygen level were evaluated using linear mixed models with Tukey’s post hoc adjustment for multiple comparisons. Statistical significance is indicated as follows: * *p* < 0.05, *** *p* < 0.001 between the specified groups. Abbreviations: mPAP, mean pulmonary arterial pressure; FiO_2_, fraction of inspired oxygen.

The pulmonary hemodynamic responses to graded hypoxia are depicted in Figure 3A,B. Under baseline normoxic conditions (FiO_2_ = 0.210), the mean pulmonary arterial pressure (mPAP) was significantly lower in Tibetan antelope (TA; 16.64 ± 3.21 mmHg) compared to high-altitude sheep (HAS; 24.41 ± 4.62 mmHg; adjusted *p* < 0.05), while no significant difference was observed between TA and low-altitude sheep (LAS; 17.70 ± 5.29 mmHg; *p* > 0.05). The baseline mPAP in HAS exceeded the established reference range for lowland sheep (15–20 mmHg), indicating elevated pulmonary vascular pressure likely due to chronic high-altitude exposure. During graded hypoxia, mPAP increased significantly from baseline in both HAS and LAS (*p* < 0.05), with a notably smaller absolute increase observed in TA (*p* > 0.05). Under conditions of both mild (FiO_2_ = 0.146) and severe (FiO_2_ = 0.125) hypoxia, the absolute mPAP differed significantly among the three groups (Figure 3A), with the highest values recorded in LAS, intermediate values in HAS, and the lowest values in TA (all pairwise comparisons *p* < 0.05; TA vs. LAS, *p* < 0.001). Compared to the normoxic baseline, mean pulmonary arterial pressure (mPAP) increased by 47% and 75% in the Tibetan Antelope (TA), 48% and 90% in the High-Altitude Sheep (HAS), and 145% and 240% in the Low-Altitude Sheep (LAS) under mild and severe hypoxic conditions, respectively (Figure 3B). The relative increase in mPAP was significantly greater in LAS compared to both TA (*p* < 0.001) and HAS (*p* < 0.05) at both levels of hypoxia, whereas the percentage increase in HAS was not significantly different from that in TA (*p* > 0.05). These findings suggest that the hypoxic pulmonary vasoconstrictor response is substantially attenuated in TA compared to LAS. Although the relative increase in mPAP in HAS was similar to that in TA, the elevated baseline mPAP in HAS resulted in higher absolute pulmonary arterial pressure during hypoxic conditions. In contrast to both sheep groups, TA maintained low absolute mPAP levels throughout hypoxic exposure, indicating the presence of adaptive mechanisms that mitigate the hypoxia-induced elevation of pulmonary arterial pressure.

### 3.4. Morphological Features of the Lung and Myocardium

**Figure 4:**
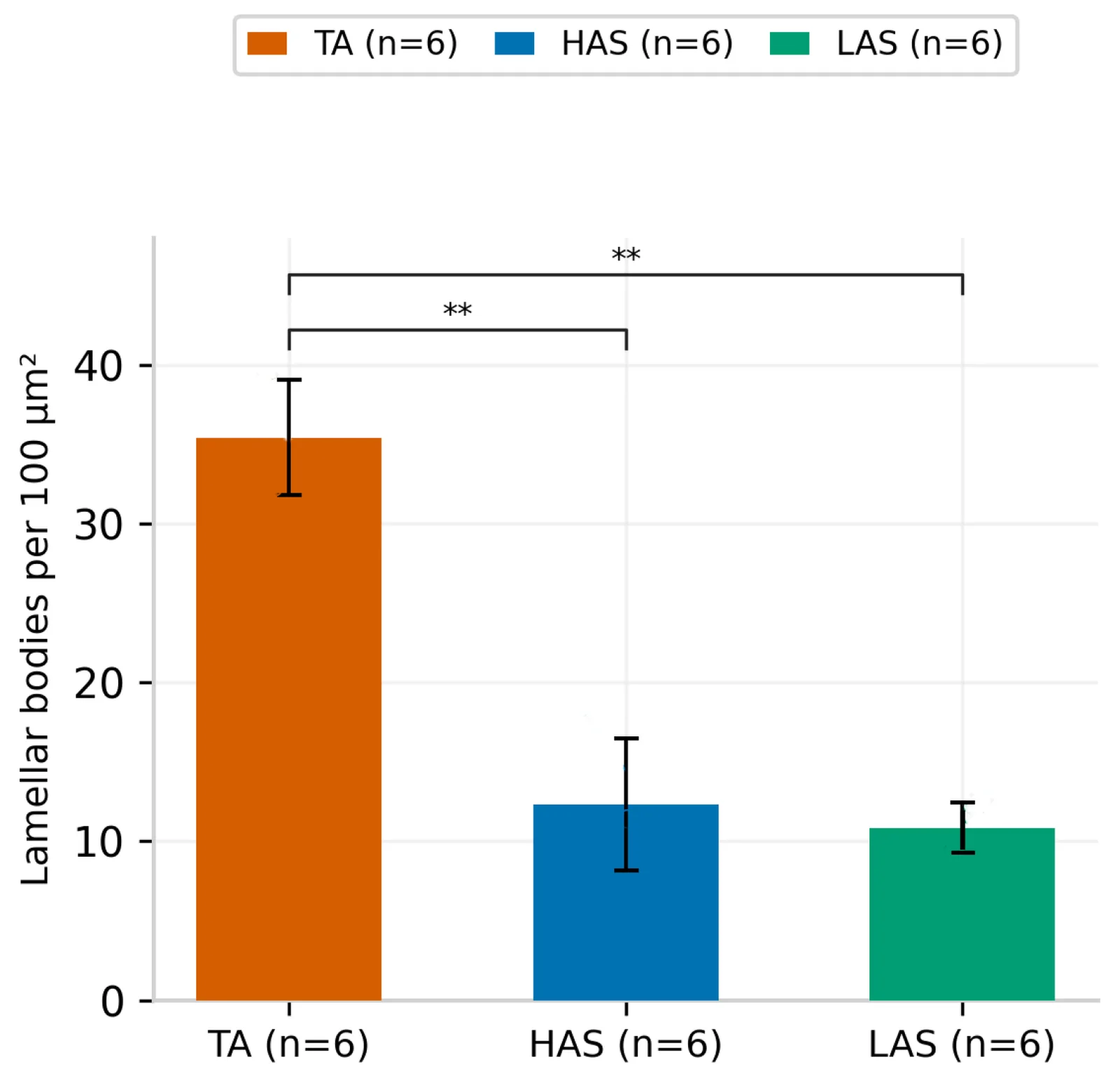
The density of lamellar bodies within alveolar type II pneumocytes in Tibetan antelope (TA), high-altitude sheep (HAS), and low-altitude sheep (LAS). The density was measured as the number of lamellar bodies per 100 μm^2^ of alveolar septal area in the right middle lung lobe of TA (*n* = 6), HAS (*n* = 6), and LAS (*n* = 6). The data are expressed as mean ± standard deviation (SD), with individual data points superimposed. Differences between groups were evaluated using a one-way analysis of variance (ANOVA) followed by Tukey’s Honestly Significant Difference (HSD) post hoc test. Statistical significance was indicated by ** *p* < 0.01 when compared to both HAS and LAS.

Ultrastructural examination revealed that alveolar type II pneumocytes in Tibetan antelope (TA) exhibited a significantly higher density of lamellar bodies compared to those in both high-altitude sheep (HAS) and low-altitude sheep (LAS), with measurements of 35.4 ± 3.6 versus 12.3 ± 4.1 and 10.8 ± 1.6 lamellar bodies per 100 μm^2^ of alveolar septal area, respectively (*p* < 0.01 for both comparisons; Figure 4). Conversely, no significant difference in lamellar body density was observed between the two sheep groups (*p* > 0.05), with only sparse lamellar bodies detected in the type II pneumocytes of HAS and LAS, as depicted in the representative transmission electron micrographs (Figure S2).

**Figure 5:**
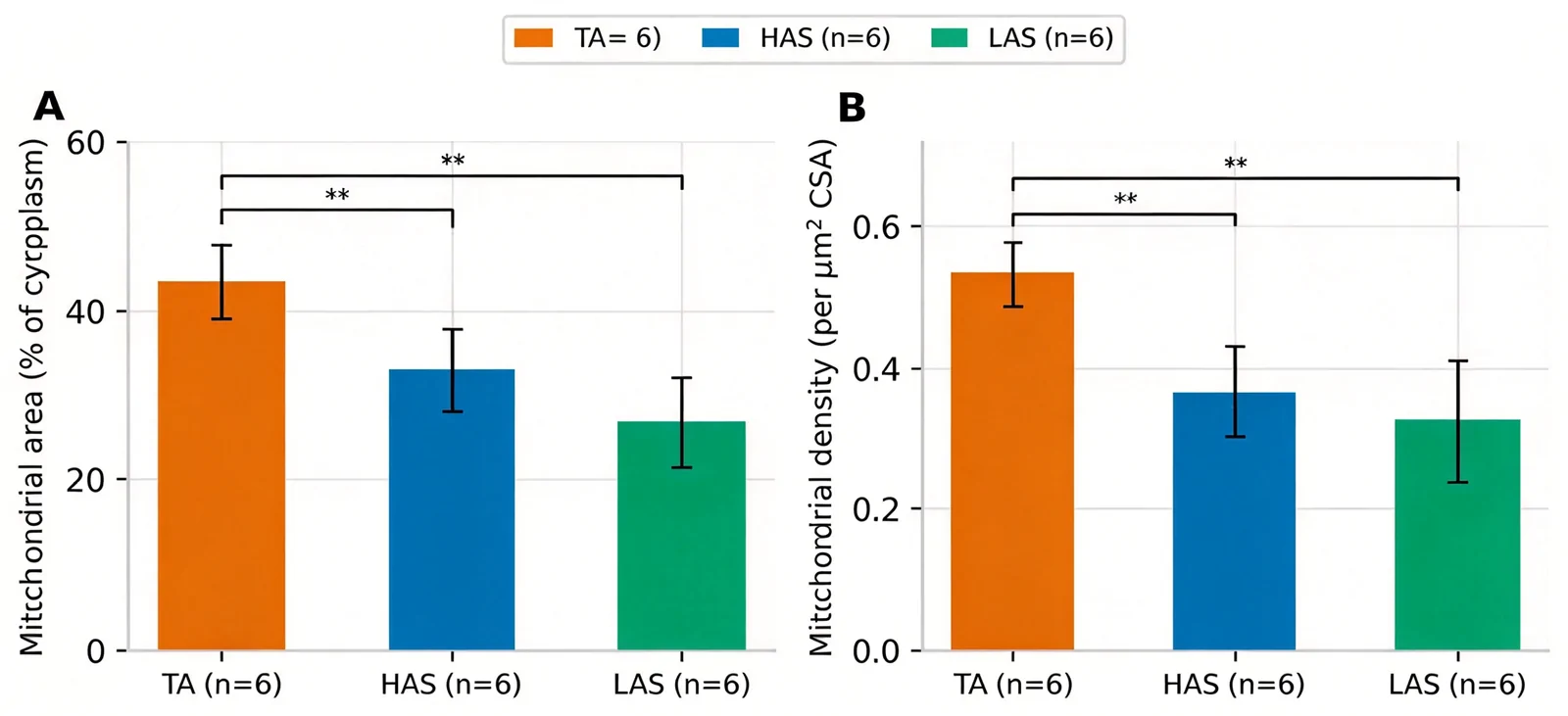
Illustrates the myocardial mitochondrial ultrastructure within the left ventricular cardiomyocytes of Tibetan antelope (TA), high-altitude sheep (HAS), and low-altitude sheep (LAS). Panel (**A**) depicts the percentage of mitochondrial area relative to the total cytoplasmic area. Panel (**B**) presents mitochondrial density, normalized to the cardiomyocyte cross-sectional area (CSA). Quantitative analyses were performed on the free wall of the left ventricle from TA (*n* = 6), HAS (*n* = 6), and LAS (*n* = 6). Data are expressed as mean ± standard deviation (SD), with individual data points superimposed. Differences among groups were evaluated using a one-way analysis of variance (ANOVA), followed by Tukey’s Honestly Significant Difference (HSD) post-hoc test. ** *p* < 0.01 indicates significance compared to both HAS and LAS. Note: CSA refers to cross-sectional area.

A quantitative morphometric analysis of left ventricular cardiomyocytes revealed that mitochondria constituted a significantly larger proportion of the cytoplasmic area in Tibetan antelope (TA; 43.4 ± 4.3%) compared to both high-altitude sheep (HAS; 33.0 ± 4.8%) and low-altitude sheep (LAS; 26.7 ± 5.2%; *p* < 0.01 for both comparisons; see Figure 5A). The density of lamellar bodies did not exhibit a significant difference between the two sheep groups (*p* > 0.05). This intergroup difference remained statistically significant even after normalization to cardiomyocyte cross-sectional area (CSA; TA, 0.53 ± 0.02; HAS, 0.37 ± 0.06; LAS, 0.33 ± 0.08 mitochondria per μm^2^ CSA; *p* < 0.01 for both comparisons; refer to Figure 5B), suggesting that the elevated mitochondrial content in TA is not solely attributable to variations in cardiomyocyte size. Representative images of mitochondrial ultrastructure are provided in Figure S3.

## 4. Discussion

**Figure 6:**
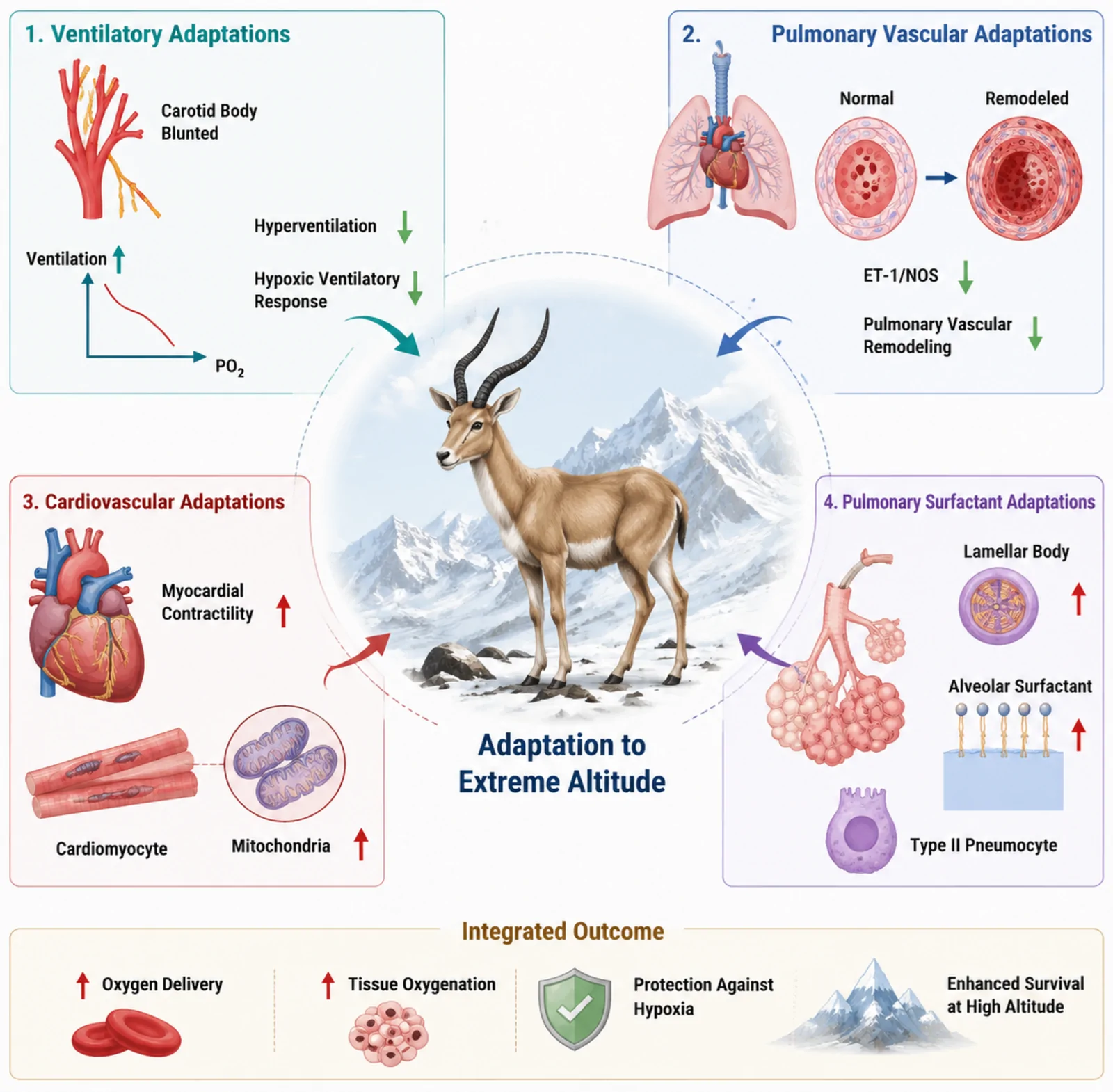
Schematic summary of key cardiopulmonary adaptive traits in the Tibetan antelope compared with sheep at high altitude.

This study is the first to comprehensively compare pulmonary hemodynamics, ventilatory regulation, vasoactive peptides, and cardiopulmonary ultrastructure between Tibetan antelopes and sheep at different altitudes. The main finding is that Tibetan antelopes have three adaptive traits: reduced hypoxic pulmonary vasoconstriction, decreased hypoxic ventilatory response, and increased alveolar lamellar bodies and myocardial mitochondria. These adaptations likely improve oxygen delivery and use at high altitudes, a result of long-term natural selection on the Qinghai-Tibetan Plateau (Figure 6).

### 4.1. Attenuated Hypoxic Ventilatory Response: A Conserved High-Altitude Adaptation

In lowland mammals, acute hypoxia induces a rapid increase in ventilation through the stimulation of peripheral chemoreceptors, specifically the carotid body. This response elevates alveolar PO_2_ but incurs an increased work of breathing and the risk of respiratory alkalosis [8,9]. In contrast, individuals native to high-altitude environments commonly exhibit a diminished HVR, which is believed to minimize the metabolic cost of breathing and prevent excessive hypocapnia, thereby safeguarding cerebral oxygenation [14,15]. Our findings indicate that TA, unlike HAS and LAS, did not exhibit a significant increase in VE under conditions of severe hypoxia (12.5% O_2_). The percentage change in VE for TA was approximately one-third of that observed in LAS under similar hypoxic conditions. This attenuated HVR in TA aligns with observations in Tibetan humans [14,15] and plateau pika [21,22,23,24], suggesting a convergent evolutionary adaptation among long-term high-altitude inhabitants.

What potential advantages might a diminished HVR offer to an animal engaged in high-speed running? Hyperventilation increases the workload of respiratory muscles, which can account for 10–15% of total oxygen consumption during intense exercise. By minimizing excessive hyperventilation, the animal may conserve oxygen for the locomotor muscles. Additionally, maintaining an elevated PaCO_2_, which showed no significant difference from that observed in sheep in our study, supports cerebral blood flow and may prevent exercise-induced cerebral hypoxia [18]. The underlying mechanism likely involves a decreased sensitivity of the carotid body to hypoxia, potentially due to alterations in ion channel expression or neurovascular coupling [25,26].

### 4.2. Attenuated Hypoxic Pulmonary Vasoconstriction: Protection Against Pulmonary Hypertension

HPV is an inherent response of the pulmonary arteries to reduced alveolar PO_2_, redirecting blood flow away from hypoxic regions to enhance ventilation-perfusion matching. However, when HPV is prolonged or intensified, it can contribute to the development of pulmonary hypertension, right ventricular hypertrophy, and high-altitude heart disease [10,11]. In this study, sheep, including both HAS and LAS acclimatized groups, exhibited a pronounced HPV response, with mPAP increasing by 90–240% depending on the severity of hypoxia. In stark contrast, TA demonstrated minimal HPV, with mPAP remaining close to baseline levels even under severe hypoxic conditions. This attenuated HPV response in TA is similar to that observed in the plateau pika [12,13] and yak [6,25], and it stands in opposition to the exaggerated HPV observed in HAPE-susceptible lowlanders [1,26].

The observed low baseline mPAP and attenuated HPV in TA were associated with lower circulating ET-1 and NOS compared to sheep. Although ET-1 reduction would decrease vasoconstrictor tone, the concurrent NOS reduction might prevent excessive vasodilation that could impair ventilation-perfusion matching. This balanced low-tone state likely underlies the attenuated HPV. The exact molecular mechanisms underlying this phenomenon merit further investigation. Such mechanisms may involve variants in the HIF pathway or endothelin receptor desensitization.

### 4.3. Ultrastructural Specializations: Lamellar Bodies and Mitochondria

Two notable morphological observations in TA include the proliferation of lamellar bodies within alveolar type II pneumocytes and the elevated density of mitochondria in cardiomyocytes. Lamellar bodies serve as storage organelles for pulmonary surfactant, which plays a crucial role in reducing surface tension, preventing alveolar collapse, and facilitating gas exchange [25,26]. The significant increase in lamellar bodies observed in TA indicates an enhanced capacity for surfactant production, which is particularly advantageous during periods of intense physical activity when the alveolar surface area undergoes rapid expansion. This observation aligns with transcriptomic data that demonstrate upregulation of surfactant proteins SP-A and SP-B in the lungs of TA [3].

The remarkably high mitochondrial density observed in TA myocardium suggests a substantial oxidative phosphorylation capacity and efficient ATP production [8]. This characteristic is essential for maintaining the elevated cardiac output required during extended periods of running at high altitudes. Conversely, the myocardium of sheep exhibits a lower density and less organized arrangement of mitochondria, which may constrain aerobic metabolism and increase dependence on the less efficient glycolytic pathway. The helical arrangement of mitochondria around myofibrils in TA is likely to enhance the rapid diffusion of ATP and minimize oxygen diffusion distances [9]. These ultrastructural distinctions provide a direct morphological foundation for the superior athletic performance of TA in hypoxic conditions. We acknowledge that the right ventricle is directly relevant to pulmonary hypertension and afterload. The left ventricle was chosen because this study aimed to investigate integrated cardiopulmonary adaptation, including myocardial oxidative capacity to support the exceptional athletic performance of Tibetan antelopes. Future comparative studies should include ultrastructural analysis of the right ventricle to fully characterize cardiac adaptation to high-altitude hypoxia.

### 4.4. Hematological Characteristics: Low Hemoglobin as an Adaptive Trait

Unexpectedly, TA individuals exhibited lower RBC and Hb concentrations compared to HAS, and these levels were comparable to those of LAS. This finding contradicts the common expectation that high-altitude natives should possess elevated Hb levels. However, excessive erythrocytosis, characterized by high Hb levels, can increase blood viscosity, elevate PVR, and contribute to chronic mountain sickness [11,16]. Unlike Andean highlanders, Tibetan populations demonstrate only modest increases in Hb, attributed to genetic variants in EGLN1 and PPARA that down-regulate erythropoiesis [16]. The reduced Hb levels observed in TA individuals may similarly function to maintain low blood viscosity, decrease right ventricular afterload, and prevent pulmonary vascular remodeling. This “low-and-thin” hematological profile appears to represent an evolved strategy to circumvent the maladaptive consequences of polycythemia [17].

### 4.5. Integration and Evolutionary Perspective

Collectively, our findings present a comprehensive understanding of high-altitude adaptation in the Tibetan antelope. At the organ level, a diminished HVR and attenuated HPV reduce energy expenditure and safeguard against pulmonary hypertension. At the cellular level, the presence of abundant lamellar bodies and mitochondria facilitates enhanced gas exchange and energy production. Systemically, low Hb levels maintain optimal blood viscosity. These traits are interdependent: low Hb levels reduce PAP, which, in conjunction with attenuated HPV, prevents high-altitude heart disease; a reduced HVR allows PaCO_2_ to remain near normal, preventing respiratory alkalosis and sustaining cerebral oxygenation; and abundant mitochondria supply the necessary ATP for prolonged running. Notably, all these characteristics are observed in TA, but not in HAS, despite the latter also residing at high altitudes. This underscores the extent to which natural selection over millennia has finely tuned the physiology of wild Tibetan antelope, surpassing the limited acclimatization or selective breeding seen in domestic sheep.

### 4.6. Study Limitations

Several limitations of this study warrant acknowledgment. Firstly, the experiments were conducted at the Golmud base (2800 m) rather than at the native altitude of TA (4300–4600 m) due to logistical constraints. Although the TA and HAS were acclimatized for only 5 days at 2800 m (vs. their native altitude >4000 m), partial de-acclimatization cannot be excluded. This short period may have led to partial loss of high-altitude traits (e.g., Hb, Hct) in HAS, making the differences between TA and HAS more conservative than under fully acclimatized conditions. However, the persistent differences in mPAP and VE responses suggest a strong genetic component. Notably, gross necropsy revealed no evidence of right ventricular hypertrophy in any group, including HAS, despite their elevated baseline mPAP. This suggests that the experimental duration and acclimatization conditions were insufficient to induce structural cardiac remodeling, and that Tibetan antelopes are protected from pressure overload due to their attenuated HPV phenotype. Future studies conducted at native altitudes are needed. Secondly, the study was unable to employ the rebreathing method to measure HVR, a standard technique in human studies, due to the impracticality of continuous SaO_2_ monitoring in unrestrained large animals. Instead, steady-state hypoxic challenges were utilized, and VE responses were reported, providing reliable comparative data; however, it is acknowledged that this approach may not fully capture the dynamic HVR slope. Thirdly, the absence of sea-level Tibetan antelopes, which do not exist, precludes the establishment of a true low-altitude control for this species, thereby limiting the ability to completely disentangle genetic adaptation from effects due to lifetime exposure. It should also be noted that within any given population, individuals differ substantially in their innate resistance to hypoxia: hypoxia-susceptible individuals are characterized by higher basal levels of HIF-1α, exaggerated pulmonary vascular and sympathetic responses, and increased oxidative stress markers, whereas hypoxia-tolerant individuals display more efficient antioxidant defenses and higher constitutive expression of protective factors such as erythropoietin, NO, and heat-shock proteins [18]. At the molecular level, low hypoxia tolerance is associated with a higher pro-inflammatory potential, reflected by elevated HIF-1α, HIF-2α, HIF-3α, and NF-κB expression in peripheral blood leukocytes [19]. These individual differences in hypoxia tolerance phenotypes may therefore have influenced the mean values reported in the present study and potentially masked true interspecies differences; future studies should stratify animals according to their hypoxia-resistance phenotypes to address this limitation [19,20]. Furthermore, while our sample sizes were sufficient for analyzing the primary outcomes, they were relatively modest for certain secondary morphometric analyses. Specifically, the LAS group included only eight animals due to limited availability, potentially diminishing the statistical power for some comparisons. Future research incorporating larger sample sizes and, ideally, measurements taken at the native altitude would enhance the robustness of the conclusions.

### 4.7. Veterinary and Clinical Implications of the Findings

From a veterinary medicine perspective, the baseline mPAP of HAS (24.4 mmHg) deserves particular attention. In domestic ruminants, a resting mPAP > 25 mmHg is considered diagnostic for pulmonary hypertension, and HAS animals in this study already hover near this threshold. This suggests that high-altitude sheep, despite centuries of residence on the plateau, may still exhibit maladaptive vascular remodeling, in contrast to TA which maintain mPAP within the normal lowland range (16–18 mmHg). This difference has practical implications for veterinary management of livestock at high altitudes: Tibetan sheep may be at higher risk for high-altitude heart disease, particularly during acute hypoxia or transport stress. The absence of pulmonary parasitic lesions in all groups—confirmed by gross and histological examination—excludes parasitism as a confounder for elevated mPAP in HAS.

Additionally, the lactate data (all within normal resting range, <1.0 mmol/L) indicate that under these experimental conditions (sedated, resting, short hypoxic exposures), none of the animals experienced systemic tissue hypoxia severe enough to trigger anaerobic glycolysis. This suggests that the observed blunted HVR and HPV in TA are not maladaptive to the point of compromising systemic O_2_ delivery under resting conditions. However, whether this holds true during maximal exercise remains to be determined.

The anesthesia protocol used in this study—ketamine-diazepam-isoflurane—is commonly employed in wild ungulate medicine and has been shown to have minimal effects on pulmonary vascular tone compared to α_2_-agonists. The addition of atropine prevented the bradycardia that would have otherwise confounded CO and PVR calculations. These methodological choices enhance the translatability of our baseline hemodynamic data to field conditions.

### 4.8. Revised Limitations

Several additional limitations from a veterinary standpoint warrant mention. First, we did not measure serum biomarkers of capture myopathy (e.g., creatine kinase, aspartate aminotransferase, or cardiac troponin I), which could have identified subclinical muscle damage that might influence hemodynamic or metabolic data. Although no clinical signs were observed, subclinical effects cannot be fully excluded. Second, the acclimatization period for TA and HAS was only 5 days, which is insufficient for complete hematological re-equilibration; however, given permit constraints and the welfare concerns associated with prolonged captivity of wild TA, this was the maximum feasible duration. Third, our morphological measurements, while standardized, are subject to tissue-shrinkage artifacts inherent to electron microscopy preparation; the normalization to septal area and myocyte area partially mitigates this issue but does not eliminate it entirely. Fourth, the use of isoflurane, while minimizing α_2_-agonist-induced vasoconstriction, may itself have mild dose-dependent effects on hypoxic pulmonary vasoconstriction; however, because all groups received identical isoflurane concentrations, any effect would be consistent across groups and unlikely to alter the relative differences observed. Fifth, we recognize that individual animals within each group may possess different innate tolerances to hypoxia, which could influence group averages and mask true inter-species differences. Future studies with larger sample sizes should consider stratifying individuals by hypoxia-tolerance phenotypes (e.g., baseline mPAP or HVR magnitude) to dissect genetic versus environmental contributions more precisely. An additional limitation is that lung tissues were immediately fixed in glutaraldehyde for ultrastructural analysis, and no fresh frozen samples were preserved. Consequently, we were unable to perform functional assays such as isolation of pulmonary artery smooth muscle cells to assess proliferative potential, or to measure key signaling molecules including Rho-kinase (ROCK), which plays a critical role in vasoconstriction and vascular remodeling. Future studies should incorporate these mechanistic analyses to further elucidate the molecular basis of the attenuated HPV observed in Tibetan antelopes. Finally, this study measured circulating effector molecules (*ET-1* and *NOS*) but did not assess upstream transcriptional regulators, specifically HIF-1α and HIF-2α protein expression or the activity of their modulators (e.g., *EGLN1/PHD2*). Unique variants of *EPAS1* and *EGLN1* have been identified in high-altitude species, including the Tibetan antelope and yak. Without analyzing these components, the question remains whether the attenuated HPV and HVR are modulated at the transcriptional, post-transcriptional, or post-translational level. Future studies incorporating HIF pathway protein expression, nuclear translocation assays, and targeted gene expression analysis are essential to resolve this question.

## 5. Conclusions

This study reveals that Tibetan antelopes have unique cardiorespiratory adaptations for high-altitude living, such as a reduced hypoxic ventilatory response and dense myocardial mitochondria. Unlike sheep, they have lower PaO_2_ and SaO_2_, suggesting their strategy focuses on balancing metabolic cost, risk, and performance rather than maximizing oxygen delivery. Their moderate hemoglobin levels and normal lactate levels indicate an energy-efficient approach. From a veterinary standpoint, Tibetan antelopes show better cardiopulmonary resilience compared to high-altitude sheep, which are more prone to hypoxic pulmonary hypertension. Our findings underscore the distinct physiological characteristics of TA, indicating that the preservation of their natural high-altitude environment is essential for maintaining these adaptive traits. Future research should prioritize the functional validation of morphological observations, evaluate exercise capacity in natural settings, and elucidate the molecular pathways underlying the attenuated hypoxic pulmonary vasoconstriction (HPV) and hypoxic ventilatory response (HVR) phenotypes.

## Figures and Tables

**Table 1:** Baseline body, pulmonary weight and hematological indicators of three animal groups (mean ± SD).

Variable	TA (*n* = 10)	HAS (*n* = 10)	LAS (*n* = 8)	*p*-Value
Body weight (kg)	37.94 ± 9.47	37.67 ± 4.67	38.00 ± 8.15	0.995
Age (years)	2.10 ± 1.10	2.50 ± 0.71	2.75 ± 1.03	0.309
Height (cm)	71.60 ± 5.44 ^b^	76.40 ± 4.25 ^a^	77.50 ± 4.25 ^a^	<0.05
Lung weight (g)	428.05 ± 25.80 ^a^	355.11 ± 83.60 ^b^	380.98 ± 60.90 ^ab^	0.042
Heart rate (beats/min)	35.20 ± 11.32 ^b^	67.75 ± 11.28 ^a^	74.75 ± 11.28 ^a^	<0.001
SAP (mmHg)	101.51 ± 11.67 ^b^	125.52 ± 11.77 ^a^	127.37 ± 13.84 ^a^	<0.001
DAP (mmHg)	73.10 ± 9.28 ^b^	90.01 ± 12.21 ^a^	85.45 ± 14.76 ^a^	<0.05
RBC (×10^12^/L)	8.29 ± 0.94 ^b^	8.81 ± 2.79 ^ab^	10.60 ± 1.49 ^a^	<0.05
Hb (g/dL)	12.23 ± 1.32 ^b^	14.13 ± 1.04 ^a^	12.91 ± 0.96 ^ab^	<0.01
Hct (%)	35.62 ± 6.72 ^b^	48.90 ± 9.74 ^a^	43.25 ± 6.33 ^ab^	<0.01
*ET-1* (mg/mL)	1.91 ± 0.98 ^c^	8.24 ± 3.03 ^a^	5.36 ± 3.84 ^b^	<0.001
*NOS* (mg/mL)	2.79 ± 2.03 ^b^	10.50 ± 4.61 ^a^	14.29 ± 6.15 ^a^	<0.001
*ET-1/NOS* ratio	0.76 ± 0.24 ^ab^	0.89 ± 0.39 ^a^	0.41 ± 0.28 ^b^	<0.05

***Notes:*** Data are presented as mean ± standard deviation (SD). TA, Tibetan Antelope; HAS, High-Altitude Sheep; LAS, Low-Altitude Sheep; SAP, systolic arterial pressure; DAP, diastolic arterial pressure; RBC, red blood cell count; Hb, hemoglobin; Hct, hematocrit; *ET-1*, endothelin-1; *NOS*, nitric oxide synthase. a–c Different superscript letters within the same row indicate significant differences between groups (one-way ANOVA followed by Tukey’s post hoc test, *p* < 0.05). Groups sharing the same superscript letter are not significantly different (*p* > 0.05). The overall *p*-value in the last column is derived from one-way ANOVA.

**Table 2:** Baseline arterial blood gas and lactate indicators (mean ± SD).

Variable/Condition	TA	HAS	LAS	*p*-Value/Note
pH	7.36 ± 0.04	7.35 ± 0.03	7.37 ± 0.04	*p* = 0.49
PaCO_2_ (mmHg)	36.8 ± 2.4	37.2 ± 2.1	36.5 ± 2.3	*p* = 0.61
PaO_2_ (mmHg)	62.3 ± 4.8 ^b^	78.5 ± 5.2 ^a^	76.9 ± 5.7 ^a^	*p* < 0.001
SaO_2_ (%)	89.2 ± 2.1 ^b^	96.4 ± 1.3 ^a^	95.8 ± 1.5 ^a^	*p* < 0.001
Lactate (mmol/L)	0.92 ± 0.21	0.88 ± 0.18	0.85 ± 0.19	*p* = 0.67

***Notes:*** Data are presented as mean ± standard deviation (SD) unless otherwise stated. TA, Tibetan antelope (*n* = 10); HAS, high-altitude sheep (*n* = 10); LAS, low-altitude sheep (*n* = 8). PaO_2_, arterial partial pressure of oxygen; PaCO_2_, arterial partial pressure of carbon dioxide; SaO_2_, arterial oxygen saturation; VE, minute ventilation; BTPS, body temperature, pressure, and saturation (condition); mPAP, mean pulmonary arterial pressure; FiO_2_, fraction of inspired oxygen. For baseline blood gas variables, the overall *p*-value in the last column was derived from one-way analysis of variance (ANOVA). For minute ventilation (VE) and mPAP measured repeatedly under three oxygen conditions, between-group differences were assessed using linear mixed models with Tukey’s post-hoc adjustment for multiple comparisons, and entries in the last column summarize the principal significant group differences reported in the main text. Superscript letters (a,b) within the same row indicate significant differences between groups; groups sharing the same superscript letter are not significantly different (*p* > 0.05).

## Data Availability

The raw physiological recording data, blood gas test records and electron microscopy image datasets generated in this study are available from the corresponding author upon reasonable request.

## Supplementary Materials

The following supporting information can be downloaded at: https://www.mdpi.com/article/10.3390/ani16203260/s1.

## Abbreviations

The following abbreviations are used in this manuscript:

**Abbreviation**	**Full Name**
TA	Tibetan antelope
HAS	High-altitude sheep
LAS	Low-altitude sheep
mPAP	Mean pulmonary arterial pressure
HPV	Hypoxic pulmonary vasoconstriction
HVR	Hypoxic ventilatory response
ET-1	Endothelin-1
NOS	Nitric oxide synthase
PaO_2_	Arterial partial pressure of oxygen
SaO_2_	Arterial oxygen saturation
PaCO_2_	Arterial partial pressure of carbon dioxide
FiO_2_	Fraction of inspired oxygen
VE	Minute ventilation
PVR	Pulmonary vascular resistance
RBC	Red blood cell
Hb	Hemoglobin
Hct	Hematocrit
