# Supplementary material for: Physiological Adaptations of Tibetan Antelopes to High-Altitude Hypoxia: Insights from Pulmonary and Vasoactive Peptide Studies
Source: Animals (Basel). 2026 Oct 16;16(20):3260. doi: 10.3390/ani16203260 (PMC13700814; doi:10.3390/ani16203260)
Supplement: Supplementary file 1 [file animals-16-03260-s001.zip › animals-4544365-supplementary.pdf]

Supplementary Figure S1

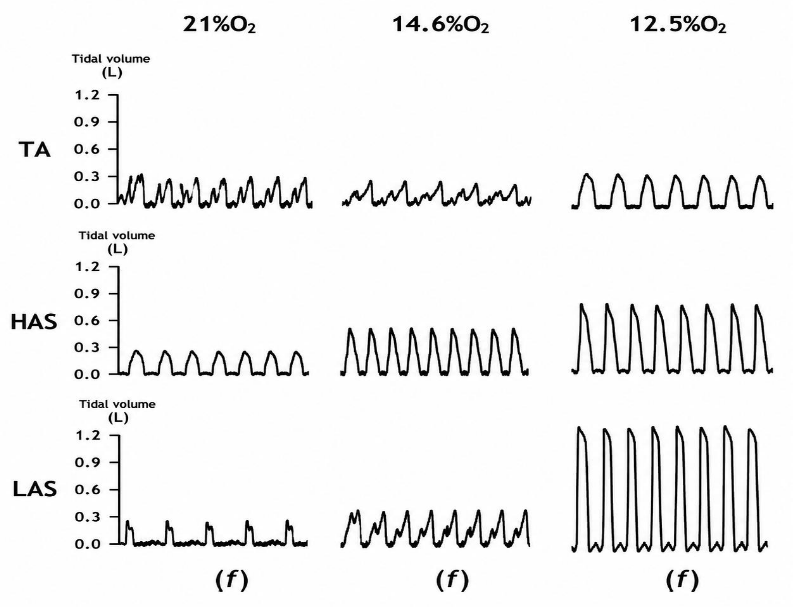

Figure S1. Representative respiratory recordings under graded hypoxia. Original respiratory waveform traces from one individual per group—Tibetan antelope (TA), high-altitude sheep (HAS), and low-altitude sheep (LAS)—under normoxia (fraction of inspired oxygen [FiO_2] = 0.210), mild hypoxia (FiO_2 = 0.146), and severe hypoxia (FiO_2 = 0.125). fR, respiratory frequency; VT, tidal volume; VE, minute ventilation. These traces are illustrative of the typical ventilatory response pattern observed in each experimental group.

Supplementary Figure S2

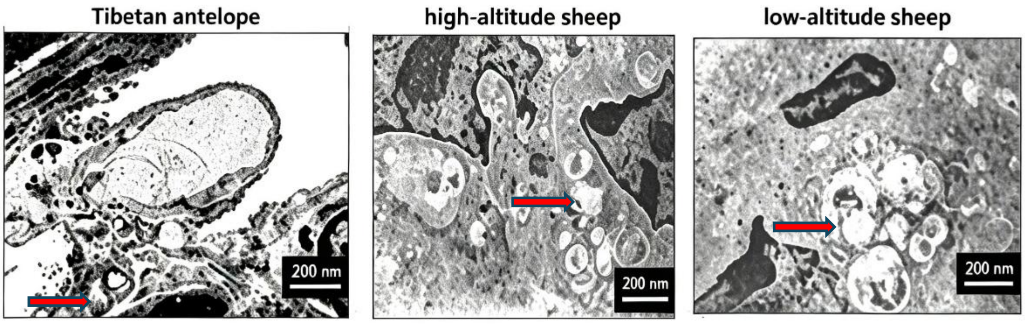

Figure S2. Representative transmission electron micrographs of alveolar type II epithelial cells and lamellar bodies in Tibetan antelope (TA), high-altitude sheep (HAS), and low-altitude sheep (LAS).

Transmission electron microscopy (TEM) images of alveolar type II pneumocytes in the right middle lung lobe. TA, HAS, and LAS. Lamellar bodies within type II pneumocytes are indicated by arrows. Lung tissue was fixed in 2.5% glutaraldehyde, postfixed in 1% osmium tetroxide, embedded in epoxy resin, and ultrathin-sectioned; sections were counterstained with uranyl acetate and lead citrate. Scale bars = 200 nm.

Supplementary Figure S3

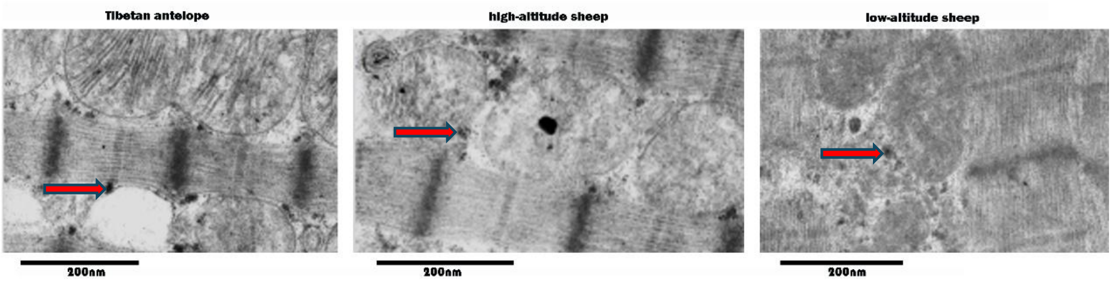

Figure S3. Representative transmission electron micrographs of left ventricular cardiomyocyte mitochondria in Tibetan antelope (TA), high-altitude sheep (HAS), and low-altitude sheep (LAS).

Transmission electron microscopy (TEM) images of cardiomyocyte ultrastructure in the left ventricular free wall. TA, HAS, and LAS. Mitochondria are indicated by arrows. Lung tissue was fixed in 2.5% glutaraldehyde, postfixed in 1% osmium tetroxide, embedded in epoxy resin, and ultrathin-sectioned; sections were counterstained with uranyl acetate and lead citrate. Scale bars = 200 nm.
